# Statistics of the Dental and Medical Professions in the United States

**Published:** 1871-09

**Authors:** 


### EDITORIAL DEPARTMENT.

*Statistics of the Dental and Medical Professions in the United States.*—Dr. Blake has collected from original sources, and with considerable labor, statistics of the dental profession from which we take the following:

There are in the United States 13,000 dentists, whose annual income is estimated at $24,000,000. Their expenses for material and rent are $6,000,000, leaving $18,000,000 as the nett income of the entire profession.

There are nine Dental Colleges, which have given to the profession 1807 graduates. The oldest of these—that of Baltimore—was founded in 1840, and has graduated 644 students. They are located, two in Boston, two in Philadelphia, while Baltimore, New York, Cincinnati, St. Louis and New Orleans have one each.

Scattered throughout our country sixty-eight Dental Associations are found in existence.

There are seven periodicals published in the interest of our profession, whose united circulation is 9,800 copies, monthly. Three of these are located in Philadelphia, and one in each of the cities of Baltimore, Buffalo, Cincinnati and St. Louis.

Dr. J. M. Toner, of Washington, D. C., has collected the following interesting statistics of the medical profession:

| | |
|---|---|
| Regular physicians | 39,070 |
| Homœpathic physicians | 2,961 |
| Hydropathic physicians | 133 |
| Eclectic physicians | 2,860 |
| Miscellaneous and unknown | 4,774 |
| Whole number of physicians of all classes | 49,798 |

This gives a ratio of 16.8 physicians to one homœopath in the whole number, and 13.1 regular physicians to one homœopath. Estimating the population of the United States in round numbers at 39,000,000, we have one regular physician to every one thousand of the population. The proportion of homœopathic physicians to the whole population would be about one in every 13,000.

